# Interdependence of Primary Metabolism and Xenobiotic Mitigation Characterizes the Proteome of Bjerkandera adusta during Wood Decomposition

**DOI:** 10.1128/AEM.01401-17

**Published:** 2018-01-02

**Authors:** S. C. Moody, E. Dudley, J. Hiscox, L. Boddy, D. C. Eastwood

**Affiliations:** a Department of Biosciences, Swansea University, Swansea, Wales; b Swansea University Medical School, Swansea University, Swansea, Wales; c School of Biosciences, Cardiff University, Cardiff, Wales; USDA Forest Products Laboratory

**Keywords:** proteome, wood decay, white rot fungus

## Abstract

The aim of the current work was to identify key features of the fungal proteome involved in the active decay of beechwood blocks by the white rot fungus Bjerkandera adusta at 20°C and 24°C. A combination of protein and domain analyses ensured a high level of annotation, which revealed that while the variation in the proteins identified was high between replicates, there was a considerable degree of functional conservation between the two temperatures. Further analysis revealed differences in the pathways and processes employed by the fungus at the different temperatures, particularly in relation to nutrient acquisition and xenobiotic mitigation. Key features showing temperature-dependent variation in mechanisms for both lignocellulose decomposition and sugar utilization were found, alongside differences in the enzymes involved in mitigation against damage caused by toxic phenolic compounds and oxidative stress.

**IMPORTANCE** This work was conducted using the wood decay fungus B. adusta, grown on solid wood blocks to closely mimic the natural environment, and gives greater insight into the proteome of an important environmental fungus during active decay. We show that a change in incubation temperature from 20°C to 24°C altered the protein profile. Proteomic studies in the field of white-rotting basidiomycetes have thus far been hampered by poor annotation of protein databases, with a large proportion of proteins simply with unknown function. This study was enhanced by extensive protein domain analysis, enabling a higher level of functional assignment and greater understanding of the proteome composition. This work revealed a strong interdependence of the primary process of nutrient acquisition and specialized metabolic processes for the detoxification of plant extractives and the phenolic breakdown products of lignocellulose.

## INTRODUCTION

Wood decay is an essential component of the carbon cycle, predominantly carried out by specialist fungi in the Agaricomycotina. White rot fungi are capable of attacking the lignocellulose matrix using reactive molecules generated by the action of a variety of oxidative enzymes, and they can subsequently metabolize all components of the wood (1). Bjerkandera adusta is a white rot fungus of the order Polyporales and an important member of the decomposer fungal community in attached branches and fallen wood of angiosperm trees, such as beech (Fagus sylvatica). In the successional community typically seen on decaying wood, B. adusta is a secondary colonizer capable of outcompeting pioneer species which begin the breakdown process (2), but it can also initiate the breakdown process independently of the primary decay community.

All biological processes are susceptible to fluctuating abiotic conditions, for example, temperature, which changes diurnally, seasonally, and over the longer term on the forest floor. An overall rise in ambient temperature has been accepted as the next phase in global environmental change, but how such changes will impact forest ecosystems remains unclear (3). As wood decay is a core process within the carbon cycle, it is vital that we understand how changes in ambient temperature impact the fungal decomposition of wood. This work investigates the composition of the B. adusta proteome as it decomposes wood and how this may be altered in response to temperature change. The baseline experimental work growing B. adusta in beechwood blocks was conducted at 20°C, as this is realistic for warm-temperate forests (4). A temperature rise of 4°C was chosen to reflect a realistic environmental temperature fluctuation and the predicted average global temperature increase (0.3 to 4.8°C) by 2100 (5). Wood decay is not only an integral part of the global ecosystem but also has potential for being industrially exploited in biorefinery (6). The way in which white rot wood decay fungi interact with wood and bring about the change in chemical composition that unlocks the carbon resource is the focus of great industrial interest, but as a complex multienzymatic process, there is still much that requires further investigation (7). The work presented here aims to explore the proteome produced by B. adusta as it decays solid beechwood blocks, in order to mimic the physical structure of the substrate encountered in natural habitats. Work has been conducted to understand the overall process of lignocellulosic decay (8, 9); now, an investigation of the proteome of individual species during colonization and decomposition of ecologically relevant substrates will provide detail on the processes underlying resource exploitation. Therefore, rather than focusing on specific proteins known to be important in the lignocellulose decay process, the nontargeted approach used here was designed to broaden the view of which proteins are produced and which pathways or processes they may represent and thereby to improve our understanding of how wood decay fungi utilize wood as both nutrient source and habitat.

## RESULTS

B. adusta was cultured on 2-cm^3^ beech (Fagus sylvatica) wood blocks for approximately 8 weeks to ensure complete colonization of the wood blocks so that hyphae were largely in log to stationary phase and exploiting the substrate, as described previously (2). Wood block cultures were then placed at either 20°C or 24°C and incubated for a further 7 days. Proteins were successfully extracted from manually chipped wood block cultures in a sodium acetate-Tween 80 buffer to maximize the isolation of the extracellular component of the proteome (10). The resultant peptide mixtures were analyzed by high-performance liquid chromatography–tandem mass spectrometry (HPLC-MS/MS).

### Annotation of the wood-associated proteome.

**FIG 1:**
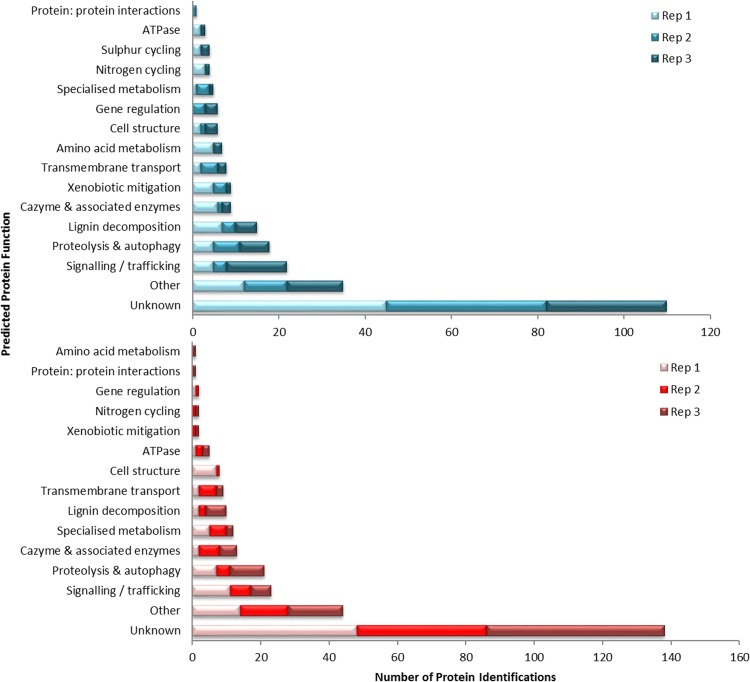
Functional classification of the B. adusta wood decay proteome of 3 biological replicates grown at 20°C (top blue bars), and 24°C (bottom red bars), based solely on BLASTP similarity comparison with the NCBI Fungal Database. Rep, replicate.

Peptide spectra were analyzed against a bespoke Mascot database composed of the B. adusta predicted proteome (available from the Joint Genome Institute [JGI] website [11]), which gave the amino acid sequence of the identified protein. The amino acid sequence was then submitted to a BLASTP search using the NCBI fungal database for functional prediction (see Table S1 for BLASTP results). Initial Mascot and BLASTP analyses of peptide spectra found high variation between replicates for both temperature treatments; of the 253 proteins identified at 20°C and 289 proteins identified at 24°C, 8 and 13 proteins were present in more than one replicate, respectively (see Table S2 in the supplemental material). At 20°C, one protein, a putative S53 protease, was identified in all replicates, and a further 7 proteins were present in two of the replicates (a fatty acid synthase, glycoside hydrolase 31 [GH31], a 3′-phosphoadenosine 5′-phosphosulfate sulfotransferase [PAPS reductase]/flavin adenine dinucleotide [FAD] synthetase, a U1 small nuclear ribonucleoprotein, a protease-like protein, a retroviral protein, and an *S*-adenosylmethionine [SAM]-dependent methyltransferase, based on protein similarity). The same putative S53 protease was identified in all three replicates at 24°C (Table S3), in addition to a putative SAM-dependent methyltransferase, a small peroxidase, and a protein of unknown function. A further 9 proteins were present in two replicates, 3 of which were also identified at 20°C (a retroviral protein, GH31, and a protease-like protein), and 6 proteins only at 24°C (a putative metallo-hydrolase/oxidoreductase, an A1 protease, actin interacting protein 3, a major facilitator superfamily transporter, a manganese peroxidase, and an uncharacterized putative transcription factor). A conserved pattern could be observed within replicates and between treatments by assessing proteins by functional classification rather than specific peptide presence (Fig. 1). Proteins characterized as having signaling/trafficking and proteolysis and autophagy functions were well represented in all replicates and at both temperatures (22 and 18 proteins, respectively, at 20°C and 23 and 21 proteins, respectively, at 24°C). Proteins involved in carbohydrate metabolism and oxidative decomposition of lignin were also present in all samples at both temperatures (9 and 15 proteins, respectively, at 20°C and 5 and 6 proteins, respectively, at 24°C).

**FIG 2:**
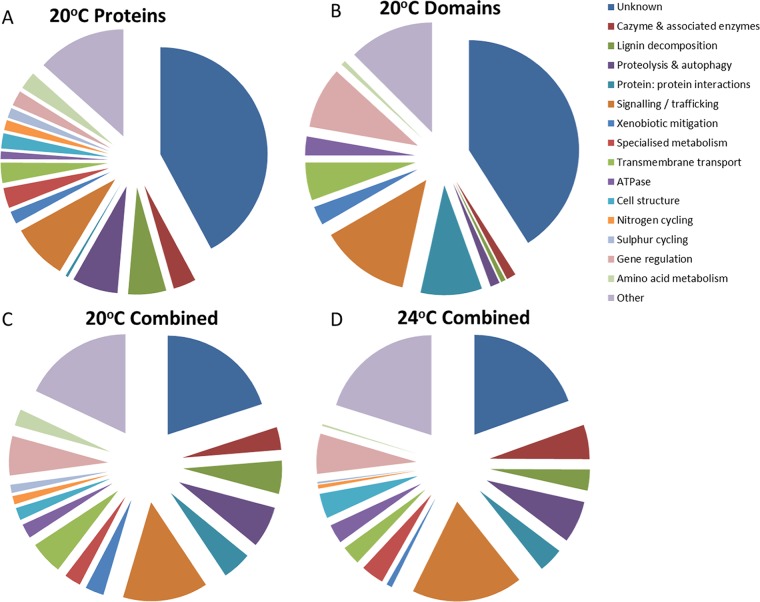
(A) The total proteins from 20°C identified by BLASTP; (B) the domain functions identified by CDD/SPARCLE in the proteins of unknown function from panel A; (C) the combination of panels A and B and hence, the total number of functional identifications made at 20°C; (D) the same combined total of proteins and domains identified at 24°C.

As our nontargeted approach aimed to identify global functional responses to substrate utilization, the total combined proteome of all three replicates per temperature was used in the subsequent analyses to give the broadest coverage of the proteins being produced by B. adusta under these conditions. (Individual proteome charts of each replicate are included in Fig. S1 to S6.) Of the 253 proteins identified across all three biological replicates at 20°C, almost 60% were annotated using BLASTP, while the remaining 40% had no known function, mostly matching hypothetical proteins from other wood decay fungi in the NCBI fungal database (Fig. 2A).

Using the CDD/SPARCLE domain analysis function (12) on NCBI and InterPro, a function was inferred for over half of the unknown proteins (Fig. 2B). In the total combined proteome, taking both known protein functions and inferred domain functions into consideration for all three biological replicates, over 75% were assigned functions (Fig. 2C), demonstrating the value of deeper analysis in defining putative functional profiles in proteomic data sets. The remainder either had no identifiable domains or contained only domains of unknown function (DUFs). The combination of BLASTP and domain analysis suggested a functional profile at 24°C for the combined proteome of 289 identified proteins (Fig. 2D) that was remarkably similar to that at 20°C (Fig. 2C).

The function most commonly suggested at 20°C at the domain level was signaling/trafficking, followed by gene regulation and protein-protein interactions (Fig. 2B). The domains predicted to be involved in signaling/trafficking included WD40; PX (Phox); and pleckstrin homology-glucosyltransferases, Rab-like GTPase activators, and myotubularin (PH-GRAM) domains (13, 14). Proteins potentially involved in protein-protein interactions contained domains such as broad-complex, tramtrack, and bric-à-brac/poxvirus and zinc finger (BTB/POZ) (15) or a tetratricopeptide repeat (TPR) (16). The number of proteins thought to be involved in gene regulation also increased (Fig. 2B compared to A). This was due to several domains known to function as part of transcriptional regulators, including some zinc binding domains (17), fungal transcription factor (TF) domains (18), and RNA recognition motif (RRM) domains (19).

As at 20°C, functional analysis based on domain reduced the number of unknown function proteins at 24°C from nearly half to approximately one-fifth, with signaling-associated domains having expanded the most. The increase in signaling/trafficking identifications at this temperature was attributable to domains such as WD40 (Trp-Asp repeats), SNF7, and several different guanine nucleotide-binding protein (G protein)-associated domains (20). The putative gene regulation proportion increased as a result of the identification of domains thought to be involved in transcription, such as Drosophila Eph kinase (DEK), switch/sucrose nonfermentable, complex B (SWIB), helix-loop-helix, and the basic leucine zipper domain (21).

### Signaling, trafficking, and transmembrane transport.

Proteins involved in signaling and trafficking from the extracellular space to the cell interior, and within the cell itself, formed a significant proportion of the proteins identified at both temperatures (Fig. 1). The signaling mechanisms employed by B. adusta included putative kinases, which showed a difference between temperatures; proteins with similarity to serine threonine kinases predominated at 24°C (6 kinases out of a total of 10), whereas a more diverse array of kinases were found at 20°C, with the proportion of putative serine threonine kinases dropping (4 out of 12). At 20°C, proteins with similarity to active transmembrane pumps of the ABC transporter family and major facilitator superfamily (MFS) transporters were predominant (22). At 24°C, the profile of the identified putative transporters changed slightly. Putative MFS transporters were again the most commonly detected type but were joined by several transporters known to be important for efflux, such as the multidrug resistance protein (MRP) transporter (which is part of the ABC superfamily) (Bjead1_1∣29795∣fgenesh1_pg.18_#_59). Another putative ABC transporter (Bjead1_1∣115643∣e_gw1.17.334.1) and a Cornichon transporter domain (Bjead1_1∣119556∣e_gw1.28.14.1) (23) were also identified at 24°C. There was little difference between 20 and 24°C in the number of transporter proteins/domains identified, suggesting that this function is essential for growth at both temperatures.

### ATPases.

**FIG 3:**
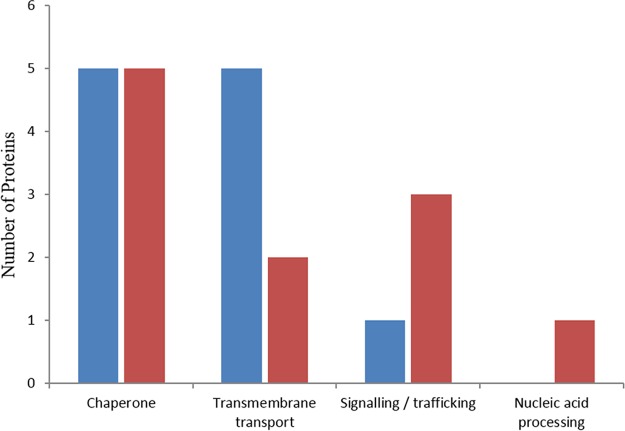
The number of proteins identified at 20°C (blue) and at 24°C (red) with putative ATPase activity and the range of functions they perform, based on NCBI BLASTP matches and domain analysis.

The energy-generating decomposition of ATP by ATPases was well represented in both proteins and domains. The amino acid sequences of the proteins enabled a more precise classification showing involvement in a variety of pathways and processes (Fig. 3 and Table S4).

The most commonly identified ATPase was a group containing the Walker A and B motifs along with a P-loop NTPase domain, showing identity with ATP-dependent Clp proteases. They are not thought to act as proteases but are characterized as ATPases associated with diverse cellular activities (AAA-ATPases), which have a role in rescuing aggregated proteins and chaperoning correct folding (24). The transmembrane transport ATPases represent a range of functions, including ABC transporters at both temperatures, P-ATPase (at 20°C) for inorganic ion transport (Bjead1_1∣105428∣e_gw1.3.1027.1), and F-ATPase (24°C) for proton transport (Bjead1_1∣28469∣fgenesh1_pg.11_#_234). The ATPases associated with signaling are predominantly those involved in cell cycle control.

### Nutrient acquisition.

**FIG 4:**
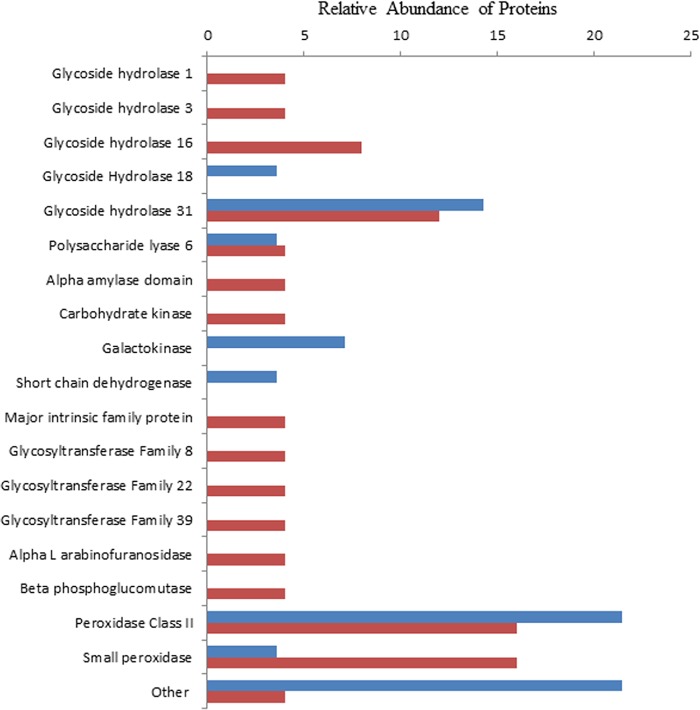
Relative abundance of proteins associated with lignocellulose decomposition at 20°C (blue) and 24°C (red) shown as a percentage of the total number of proteins identified as involved in lignocellulose decomposition (22 and 25 proteins at 20°C and 24°C, respectively).

The two temperature conditions produced different protein profiles putatively associated with lignin decomposition and carbon acquisition (Fig. 4 and Table S5).

At 20°C, the sugar-metabolizing protein profile was dominated by the carbohydrate-active enzyme (CAZyme) glycoside hydrolase 31 (GH31) (known to act on glucose, galactose, mannose, and α-xylose, among others), but few other cellulolytic enzymes were identified. Proteins with similarity to class II peroxidases were the most commonly identified, suggesting that at this temperature, the fungus might primarily use free radical attack to decompose lignin. There were also a range of other enzymes putatively associated with lignin decomposition, such as copper radical oxidase (Bjead1_1∣41582∣fgenesh1_kg.17_#_199_#_Locus11735v1_medCvg15.6s), a galactose oxidase domain-containing protein (Bjead1_1∣103129∣e_gw1.1.35.1), and cupredoxin (Bjead1_1∣109531∣e_gw1.7.863.1) (25). The protein profile at 24°C was more diverse for those proteins with similarity to CAZymes, with a far greater range of GHs appearing to be present: GH1 and GH3 (which metabolize cellulose and hemicellulose-derived sugars) and GH16 (involved in hemicellulose metabolism). The other enzymes possibly associated with lignin decomposition which were found at both temperatures included putative glyoxylate-associated proteins (a putative glyoxylate reductase, Bjead1_1∣34067∣fgenesh1_kg.1_#_869_#_Locus2702v3_medCvg78.2s, and a citrate synthase domain, Bjead1_1∣25154∣fgenesh1_pg.2_#_341). Glyoxylate is thought to be a key component of H_2_O_2_ generation by peroxidases (26). A small protein (Bjead1_1∣121766∣e_gw1.37.43.1) was identified at both temperatures, which matches class II peroxidases in the NCBI database. At 180 residues, it was half the length of a normal fungal peroxidase and appeared to lack any of the expected binding sites (heme, Ca^2+^, and Mn^2+^). It did, however, contain DUF3415, recently renamed the peroxidase_ext domain.

It is noteworthy that although enzymes specifically dedicated to nitrogen metabolism were few, at both temperatures, a significant proportion (6.7%) of the proteome was predicted to be involved in proteolysis and autophagy (Fig. 2C and D).

### Specialized metabolism and xenobiotic mitigation.

**FIG 5:**
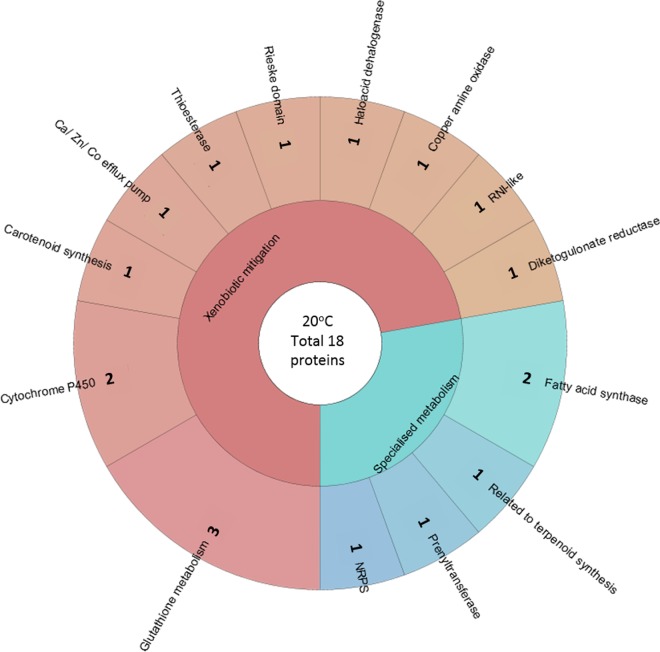
The relative abundance of each type of protein as a proportion of the total number of proteins identified as involved in specialized metabolism and xenobiotic mitigation at 20°C. RNI-like, reactive nitrogen intermediate-like protein; NRPS, nonribosomal peptide synthase.

The proteins involved in specialized metabolism at 20°C included multidomain proteins, such as putative fatty acid synthase and nonribosomal peptide synthase (Fig. 5 and Table S6). The putative prenyltransferase identified (Bjead1_1∣119726∣e_gw1.28.175.1) may be involved in endogenous terpenoid synthesis (as other enzymes from this specialized metabolic process were also found) (27), or they may prenylate plant-derived compounds (28). Two proteins identified at 20°C and 24°C have significant homology to known P450 families CYP512 (Bjead1_1∣104989∣e_gw1.3.831.1) and CYP5139 (Bjead1_1∣119120∣e_gw1.26.116.1), respectively, both of which were labeled as xenobiotic mitigation, as they are known to be involved in polyaromatic breakdown and degradation of plant extractives (29).

Proteins putatively involved in glutathione metabolism formed the largest group of those engaged in xenobiotic mitigation at 20°C, with the majority being identified as glutathione *S*-transferases, which are thought to play a role in protection from the oxidative environment caused by free radical lignin attack (30). The Rieske domain-containing proteins identified are a component of aromatic ring hydroxylating dioxygenases, indicative of a role in the degradation of polycyclic aromatic hydrocarbons.

**FIG 6:**
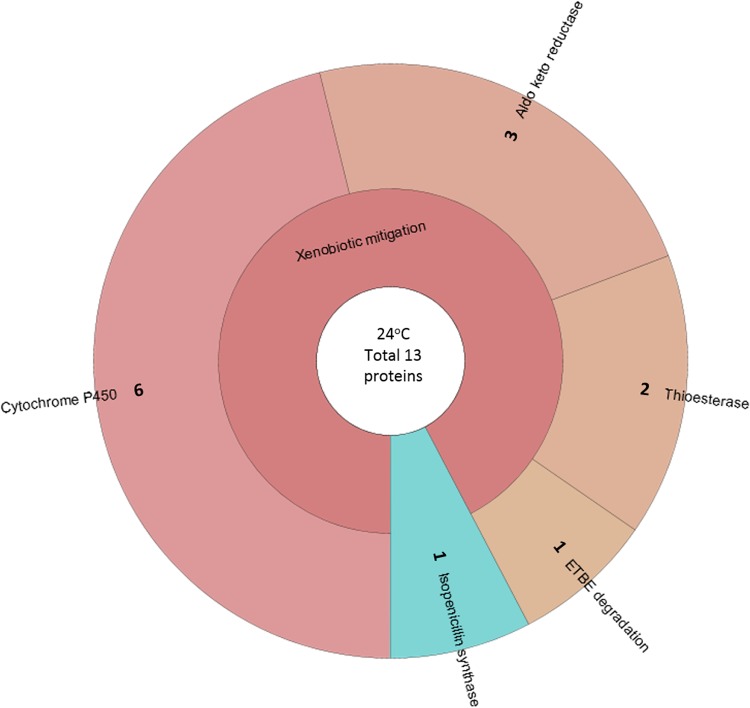
The relative abundance of each protein or process as a proportion of the total number of proteins identified as involved in specialized metabolism and xenobiotic mitigation at 24°C. 2OG-Fe(II)-dependent dioxygenase, 2-oxoglutarate Fe(II)-dependent dioxygenase; ETBE degradation, ethyl *tert*-butyl ether degradation protein.

**FIG 7:**
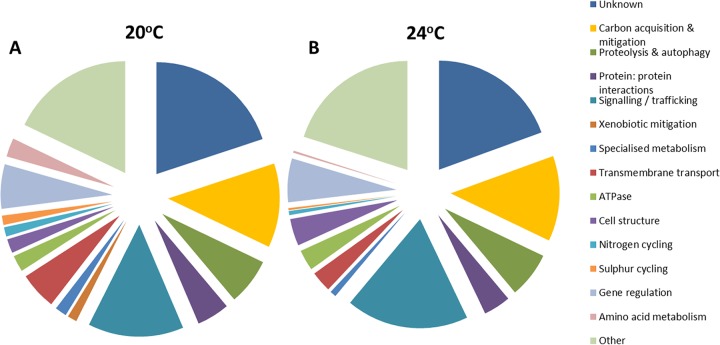
The total predicted proteome at each temperature, highlighting the combined proportion of the proteome dedicated to carbon acquisition and mitigation of xenobiotics produced during lignocellulose decomposition (yellow segment) at 20°C (A) and 24°C (B), as a percentage of the total extracellular proteome.

Cytochrome P450 proteins were present in samples from both temperatures but appeared to dominate the protein profile at 24°C (Fig. 6 and Table S6). The cytochrome P450 families represented at 24°C were CYP5035, CYP5139, CYP5144, CYP5150, CYP63, and CYP5158. Aldo/keto-reductases (AKRs) are redox enzymes with a variety of functions, none of which were specifically defined by the NCBI matches generated in this data set. Other enzymes identified at 24°C included a protein with a putative 4-hydroxybenzoyl coenzyme A (CoA) thioesterase domain (Bjead1_1∣70747∣estExt_fgenesh1_pg.C_110132) potentially involved in the degradation of benzoates, which have been associated with lignin decay; and a protein with a high degree of similarity to an ethyl *tert*-butyl ether degradation protein from Moniliophthora roreri (see Discussion for further details). The only protein putatively involved in specialized metabolism (Bjead1_1∣101126∣e_gw1.1.445.1) was identified as an isopenicillin synthase. While there is no evidence of B. adusta producing a penicillin, this protein may fulfill a similar catalytic role in specialized metabolite biosynthesis. Combining the data for the proteins putatively characterized by carbohydrate metabolism, lignin decomposition, and those xenobiotic mitigation enzymes involved in the degradation of plant extractives or detoxification of lignin breakdown products allowed a prediction of how much energy B. adusta expends acquiring carbon and mitigating against the reactive oxygen species (ROS) and toxic phenolics produced during this process (Fig. 7 and Table S7).

At both temperatures, a total of 12% of the predicted proteome was involved in carbon acquisition and xenobiotic mitigation, suggesting that these twinned functions are highly prioritized during fungal growth and survival in wood, although further experimentation is required to confirm a direct role of these proteins in wood decomposition. The only functional group that appeared to have a greater proportion was that involved in signaling, which is presumed to be important for a microbe responding to an oxidative potentially toxic environment.

## DISCUSSION

Lignocellulose decomposition is central to the lifestyle of white rot fungi, such as B. adusta. In common with other wood decay fungi, the woody matrix is a potential hazard (due to the presence of toxic plant extractives), a habitat, and a nutrient source. Substrate exploitation requires mitigation against plant extractives (31), nutrient acquisition via free radical attack on the phenylpropanoid lignin polymer (32), and concurrent mitigation against both ROS and toxic lignin derivatives (33, 34). Our results suggest a strong interdependence between nutrient acquisition and xenobiotic mitigation, with the need for carbon being balanced by the necessary detoxification of the immediate environment.

Our experimental design used well-colonized beechwood blocks to provide a realistic 3-dimensional solid substrate to best approximate the interaction between fungus and substrate in the field. The degree of precolonization would mean that B. adusta hyphae would be exploiting the resource in the substrate in log to stationary phase prior to the application of temperature treatment, as opposed to actively colonizing via hyphal growth (35). Therefore, the differences observed between the treatments are most likely a consequence of temperature effects on substrate utilization by the fungus. Higher incubation temperature is associated with an increased wood decomposition rate and the stimulation of enzyme activity in fungi (36–38), although none of these studies used B. adusta. While the Arrhenius equation generalizes that enzyme activity doubles with every 10°C increase in temperature, the wood block density losses caused by B. adusta at 20°C and 24°C are similar (35). The effects of small temperature changes on the expression level of transcripts or proteins specifically associated with woody substrate decomposition are not widely reported. An increase in temperature from 15°C to 18°C did not affect decay enzyme activity (including cellobiohydrolase, β-glucosidase, peroxidase, and β-xylosidase) in Resinicium bicolor and Phanerochaete velutina (39). However, Hietala et al. (40) showed differential transcript levels of glycoside hydrolase (GH3, GH5, and GH15), oxalic acid synthesis, alcohol oxidase, and polyphenol oxidase genes in Postia placenta when cultured at 23.5°C and 30°C, with differing profiles on soft and hard wood.

An important consideration is the high variation in the specific proteins identified between replicates and the apparent conservation in functional classification. Solid wood is a complex substrate which is chemically altered as the fungus decomposes the lignocellulose composite and deteriorates macro and cellular structures. The wood blocks used were cut from functional sapwood along a similar orientation to reduce between-sample variation. However, the depth of wood from which they were cut varied, and some structural and composition differences would be expected. It is not known to what extent such variation between wood blocks might influence the proteomes observed between samples. It might be expected that B. adusta responds to the dynamic process of wood decay with an adaptive proteome, resulting in the high variation in the specific proteins observed in this study. Common processes, however, were recorded by carrying out a functional characterization of the proteome, which may indicate a degree of functional redundancy in the proteins produced that bring about substrate decomposition. This is not a new phenomenon, as functional redundancy has been noted previously to be widespread at both at the individual protein and systems levels (41). It has been suggested that while proteins may have different but overlapping functions within metabolic networks, a by-product of this is robustness to changing environmental conditions (42). It has also been shown that functional redundancy is most commonly a property of unrelated genes rather than genes generated by duplication events (43), which correlates with the patterns seen in our data. The protein profile from our data suggested that ligninolytic activity via free radical production was dominant at 20°C, with a possible shift to decomposition of the carbohydrate components of lignocellulose at 24°C. The lack of cellulolytic GH enzymes at both temperatures was unexpected and might indicate that certain enzymes with high substrate affinity (particularly those with the carbohydrate binding module 9 motif) were not isolated despite being present; this might be further compounded by the use of cellulose-based filters in protein preparation methodology. More disruptive extraction procedures or targeted enzyme activity measurement could be included in future work to confirm the presence of such enzymes.

A range of enzymes potentially dedicated to xenobiotic mitigation was seen at both temperatures, possibly centered around cytochrome P450-mediated xenobiotic breakdown and several systems for defense against ROS generated during free radical attack of lignocellulose. Few enzymes that could be described as directly associated with nitrogen metabolism were identified, but proteins associated with putative proteasome and autophagy activity represented almost 7% of the proteome at both temperatures. These catabolic processes have previously been associated with fungi recycling macromolecules during nitrogen limitation (44, 45) and may further indicate the nutritionally stringent conditions that B. adusta experiences growing in wood. The data presented highlight groups of proteins that may be targeted in future functional studies to determine temperature effects on substrate decomposition in B. adusta.

Rather than focusing on specific groups of proteins known to be involved in wood decay, the nontargeted approach used here enabled the identification of previously unconsidered proteins produced by B. adusta during active growth on wood. While several wood decay fungal proteomes are now available (for example, see references 46–48), to our knowledge, this is the first study to conduct domain-level analysis for functional prediction of all isolated proteins. As domains are the evolutionary basis for protein structure and function (49), this enabled greater functional prediction and enriched our knowledge base of the potential of proteins involved in wood-colonizing fungi. In common with other proteomic studies of wood decay fungi (e.g., reference 50), our data revealed the production of a considerable number of B. adusta-specific proteins of unknown function which generated no match in NCBI. There were significant differences in the number of these unique proteins identified under the two experimental conditions (24 proteins at 20°C and 42 proteins at 24°C). These proteins cannot be regarded as hypothetical, as they have been expressed. Their production during the decomposition of beechwood suggests a role in fungal activity during wood decay, and they are certainly worthy of further study.

The analysis of the proteome highlighted a number of proteins that have similarity to enzymes with specific functions potentially relevant to decay but have not been widely reported previously. For example, the presence at both temperatures of a putative small peroxidase (Bjead1_1∣121766∣e_gw1.37.43.1) containing the peroxidase_ext domain but lacking the typical ion binding sites was interesting (Fig. 4). The peroxidase_ext domain is typically found near the C terminus as an extension to a heme peroxidase domain in some fungi, predominantly wood decay or plant-associated fungi. Of 446 examples in UniProt that contain the peroxidase_ext domain, 436 are from Agaricomycetes (basidiomycetes) and 10 are from Leotiomycetes (ascomycetes). To put that in perspective, UniProt has 9,945 fungal proteins with the general peroxidase domain present. Of those, 6,462 proteins are from ascomycetes and 2,973 proteins are from basidiomycetes. It appears that the occurrence of the peroxidase_ext domain is biased toward the basidiomycetes and not just conserved as part of the peroxidase architecture. This suggests a conserved function, even if it is currently unknown. Peroxidases without the typical conserved binding sites have been reported in wood decay fungi previously (51), which suggests that the small B. adusta peroxidase may not be unique.

In addition, the presence of a putative ethyl *tert*-butyl ether (ETBE) degradation protein (Bjead1_1∣106545∣e_gw1.4.377.1) was interesting (Fig. 6). ETBE is a known petroleum additive and is subject to bioremediation via Rhodococcus among other bacterial species (52). ETBE is unlikely to be a breakdown product of lignin, and ETBE degradation is not known to be a function of fungi, but NCBI BLASTP of the B. adusta protein generates numerous matches to hypothetical proteins, almost all from wood decay fungi. At the time of searching, only one other species with an ETBE degradation protein annotated was found: Moniliophthora roreri, with cocoa frosty pod rot (this had 85% coverage, 34% identity, and an E value of 1e−20 with the B. adusta protein). Overall, this suggests that an ETBE degradation protein (with unknown xenobiotic catabolic activity) may be well conserved in plant-associated saprotrophic fungi, but it has yet to be characterized.

Primary metabolism (nutrient acquisition), ligninolysis, and specialized metabolism (production of specialized metabolites and breakdown of xenobiotics) appear to be interdependent processes during growth on wood. Some putatively described enzymes identified in this study may have a dual purpose, fulfilling roles in both nutrient acquisition and xenobiotic mitigation. For example, the putative copper amine oxidase identified at 20°C (Bjead1_1∣105267∣e_gw1.3.846.1) is capable of oxidizing xenobiotic amines, with the concurrent production of ammonia and hydrogen peroxide; it therefore represents an overlap between primary metabolism (lignin decomposition and nitrogen metabolism [53]) and xenobiotic mitigation. A possible role of the putative aldo/keto-reductases (AKRs) identified at 24°C is as part of the vanillin reduction pathway (54). Vanillin is a toxic phenolic aldehyde produced as an intermediate during lignin decomposition, which can be reduced by AKR to the less harmful vanillyl alcohol. They were included here in xenobiotic mitigation but could equally be involved in specialized metabolism, or even primary metabolism. The proteins with similarity to cytochrome P450 families identified were putatively associated with xenobiotic detoxification of specialized plant flavonoids and resins, as well as xenobiotic mitigation of the polyaromatic compounds produced as a result of lignin decomposition (29), thus potentially demonstrating involvement in both primary and specialist metabolism. CYP5144 and CYP63 (expressed at 24°C) (Bjead1_1∣100963∣gw1.17.624.1 and Bjead1_1∣107364∣e_gw1.5.324.1, respectively) and CYP512 (expressed at 20°C) (Bjead1_1∣104989∣e_gw1.3.831.1) are known to be upregulated during wood decay in other species and are exclusively found in wood decay fungi (reference 29 and references therein), suggesting a specialist role in lignocellulose decomposition.

We conclude that during fungal wood decay, the primary food resource may become a source of toxic phenolic derivatives and harmful ROS as lignocellulose depolymerization occurs, making the need for efficient and immediate xenobiotic mitigation a priority. This interdependence and coordination in the proteome may be key to the survival of decay fungi colonizing a complex lignocellulosic food source.

## MATERIALS AND METHODS

### Culture conditions.

Beechwood cubes of 2 cm^3^ were autoclaved 3 times, soaked in sterile water, and added to 0.5% malt extract (5 g · liter^−1^ malt extract, 20 g · liter^−1^ agar no. 2; Lab M, UK) plates colonized by B. adusta (BaSS1). The wood blocks were incubated for approximately 8 weeks at 20°C in the dark. The external fungal biomass was scraped from the surface and the block placed on perlite moistened to −0.012 kPa at 20°C or 24°C for 7 days. At the end of the incubation, the blocks were frozen under liquid nitrogen, freeze-dried, and stored at −80°C. This was performed in triplicate for each temperature treatment.

### Protein extraction and peptide preparation.

The protein extraction process was adapted from Zeytuni and Zarivach (16) and is summarized here. It was designed to minimize cell breakage and therefore increase bias for the extracellular proteomic portion, which was of most interest. The colonized block was manually chipped into sections approximately 15 mm by 3 mm by 2 mm, using a 2-cm steel blade chisel, and then placed in 50 ml of 50 mM sodium acetate buffer (pH 5.2) with 0.05% Tween 80 and incubated overnight at 4°C and 150 rpm. The buffer was then strained through sterile wool, Whatman filter paper (no. 1), and finally through a 0.2-μm-pore-size filter. The liquid was then concentrated using Amicon centrifugal filter tubes (Merck Millipore) with a 3,000 molecular weight cutoff (MWCO) and centrifuged at 3,500 × *g* and 4°C. The concentrate was mixed with 4 volumes of ice-cold acetone before centrifugation at 10,000 × *g* for 1 h. The supernatant was removed, and the isolated protein pellet was resuspended in 1,000 μl of 50 mM sodium acetate buffer (pH 5.2) with 20 μl of protease inhibitor cocktail (Promega) and stored at −80°C. The proteomic extraction was performed on biological triplicates (3 separate colonized wood blocks). The total extracellular protein samples were separated initially on both 12% acrylamide-bisacrylamide standard SDS-PAGE gels and on 18% low-molecular-weight gels. The gels were silver stained prior to band excision (using the Pierce silver staining kit; Thermo Fisher Scientific). After destaining, each band was treated with trypsin digestion overnight, followed by peptide extraction into 0.5% formic acid in 70:30 water-acetonitrile. Samples were then dried and stored at −20°C. Each sample was rehydrated in 25 μl of 0.1% trifluoroacetic acid (TFA) prior to analysis.

### Mass spectrometry and protein identification.

HPLC-MS/MS was carried out using electrospray ionization (ESI) ion trap mass spectrometry. Full experimental details can be found in the supplemental material (information S1). The B. adusta predicted proteome (11) was downloaded from the JGI website (http://genome.jgi.doe.gov/Bjead1_1/Bjead1_1.home.html) and used to construct a proteomic database for Mascot to search. This gave a database of predicted proteins, the amino acid sequence, and the genetic location, but it did not supply the protein identities. The spectra from each sample were compared against the bespoke Mascot database to identify individual proteins. During the Mascot search, a significance threshold of a *P* value of <0.05 was applied as part of the search criteria; hence, all proteins identified were included in the analysis. Furthermore, the use of a bespoke proteomic database ensured that false-positive identifications from organisms more abundantly represented within generic protein databases were eliminated. A table showing all the proteins detected by mass spectrometry, their proteome reference number (as relating to the JGI database), Mascot score, and peptide coverage are included in the supplemental material (Table S1). Each amino acid sequence was submitted to NCBI BLASTP (using a BLOSUM62 matrix), using only fungal sequences from the nonredundant protein database, with an E value cutoff of <1e−5. To characterize the proteins with unknown function, the CDD/SPARCLE domain analysis function (12) on NCBI was used to generate a map of any domains. Domain identifications on CDD had an E value cutoff of <1e−03, but each domain was also manually curated for accuracy. Each domain was then investigated using InterPro (55) to assign a putative function to the protein. In the case that more than one domain was found, each type of domain was treated as a separate entity and assigned a function.

## Supplementary Material

Supplemental material
