# Supplementary material for: Interdependence of Primary Metabolism and Xenobiotic Mitigation Characterizes the Proteome of Bjerkandera adusta during Wood Decomposition
Source: Appl Environ Microbiol. 2018 Jan 2;84(2):e01401-17. doi: 10.1128/AEM.01401-17 (PMC5752865; doi:10.1128/AEM.01401-17)
Supplement: Supplemental material [file supp_84_2_e01401-17__index.html]

Supplemental material 

# Interdependence of Primary Metabolism and Xenobiotic Mitigation Characterizes the Proteome of Bjerkandera adusta during Wood Decomposition

## Supplemental material

- Supplemental file 1 -

  Proteins found in more than one of the biological replicates at 20°C (Table S2) and 24°C (Table S3); functional proteomes of samples 1 (Fig. S1), 2 (Fig. S2), and 3 (Fig. S3) grown at 20°C; functional proteomes of samples 1 (Fig. S4), 2 (Fig. S5), and 3 (Fig. S6) grown at 24°C; genomic locations of ATPase domains (Table S4); HPLC mass spectrometry method (information S1); genomic locations of proteins predicted to be involved in carbohydrate metabolism or lignin decomposition (Table S5); genomic locations of proteins predicted to be involved in specialised metabolism or xenobiotic mitigation (Table S6); functional predictions, by temperature (Table S7).

  PDF, 947K
- Supplemental file 2 -

  Genome reference numbers, Mascot scores, and peptide coverage (Table S1).

  XLSX, 37K
