# Supplementary material for: Interdependence of Primary Metabolism and Xenobiotic Mitigation Characterizes the Proteome of Bjerkandera adusta during Wood Decomposition
Source: Appl Environ Microbiol. 2018 Jan 2;84(2):e01401-17. doi: 10.1128/AEM.01401-17 (PMC5752865; doi:10.1128/AEM.01401-17)
Supplement: Supplemental material [file AEM.01401-17_zam002188264s1.pdf]

Supplementary information: *B. adusta* proteome

Supplementary table S2. The proteins found in more than one of the biological replicates at 20°C.

**Protein ID**	**No of Reps**	**Predicted Function**
Bjead1_1|111368|e_gw1.9.361.1	3	S53 protease
Bjead1_1|117806|e_gw1.22.244.1	2	GH31
Bjead1_1|115340|e_gw1.16.506.1	2	3'-phosphoadenosine 5'-phosphosulfate sulfotransferase (PAPS reductase)/FAD synthetase
Bjead1_1|100340|gw1.20.361.1	2	Retroviral protein
Bjead1_1|127986|estExt_Genewise1.C_2_t30346	2	U1 small nuclear ribonucleoprotein
Bjead1_1|25602|fgenesh1_pg.3_#_277	2	Fatty acid synthase
Bjead1_1|109353|e_gw1.7.668.1	2	SAM dependent methyltransferase
Bjead1_1|106781|e_gw1.4.449.1	2	S53 protease-like protein

Supplementary table S3. The proteins found in more than of the biological replicates at 24°C.

Protein ID	No of Reps	Predicted Function
Bjead1_1|109353|e_gw1.7.668.1	3	S-adenosylmethionine-dependent methyltransferase
Bjead1_1|121127|e_gw1.34.256.1	3	Unknown
Bjead1_1|121766|e_gw1.37.43.1	3	Small peroxidase
Bjead1_1|111368|e_gw1.9.361.1	3	S53 protease
Bjead1_1|30628|fgenesh1_pg.23_#_131	2	Retroviral protein
Bjead1_1|26068|fgenesh1_pg.4_#_290	2	Metallo-hydrolase / oxidoreductase
Bjead1_1|100291|gw1.30.211.1	2	A1 protease
Bjead1_1|117806|e_gw1.22.244.1	2	GH31
Bjead1_1|26706|fgenesh1_pg.6_#_29	2	Actin interacting protein 3
Bjead1_1|106781|e_gw1.4.449.1	2	S53 protease-like protein
Bjead1_1|29668|fgenesh1_pg.17_#_136	2	Major facilitator superfamily transporter
Bjead1_1|118657|e_gw1.24.388.1	2	Manganese peroxidase
Bjead1_1|165166|gm1.1605_g	2	Transcription factor

Figure S1. The functional proteome of sample #1 grown at 20°C.

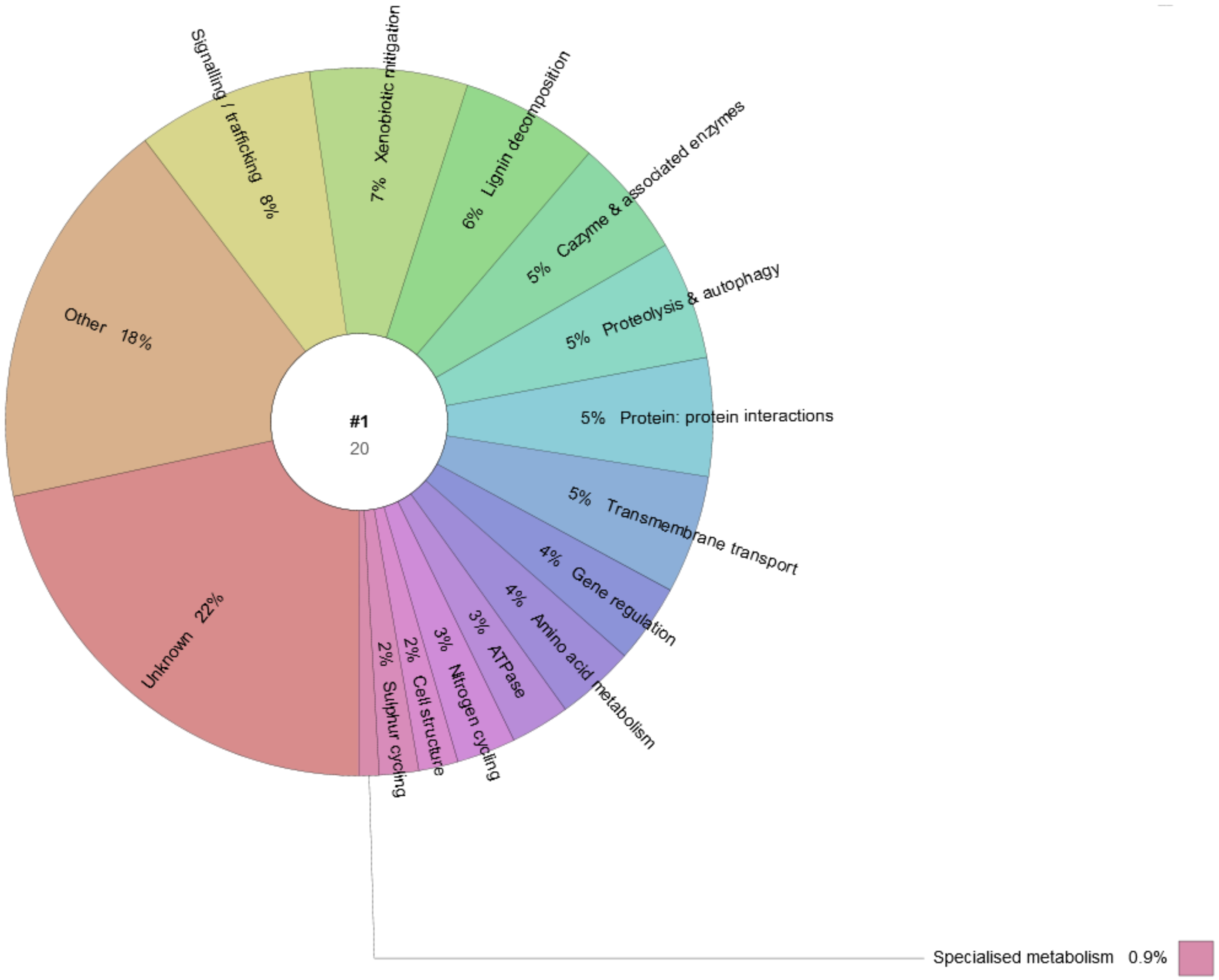

Figure S2. The functional proteome of sample #2 grown at 20°C.

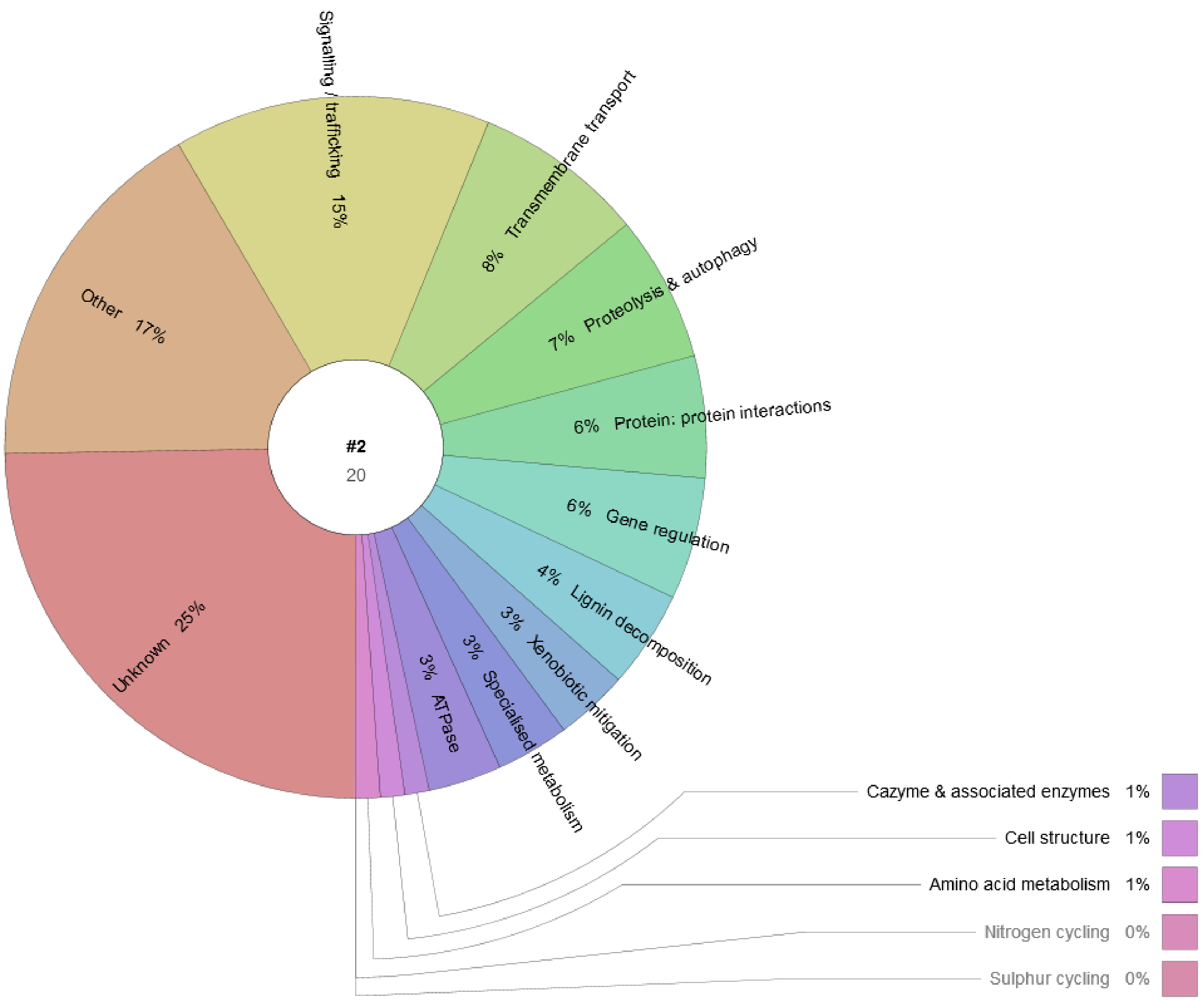

Figure S3. The functional proteome of sample #3 grown at 20°C.

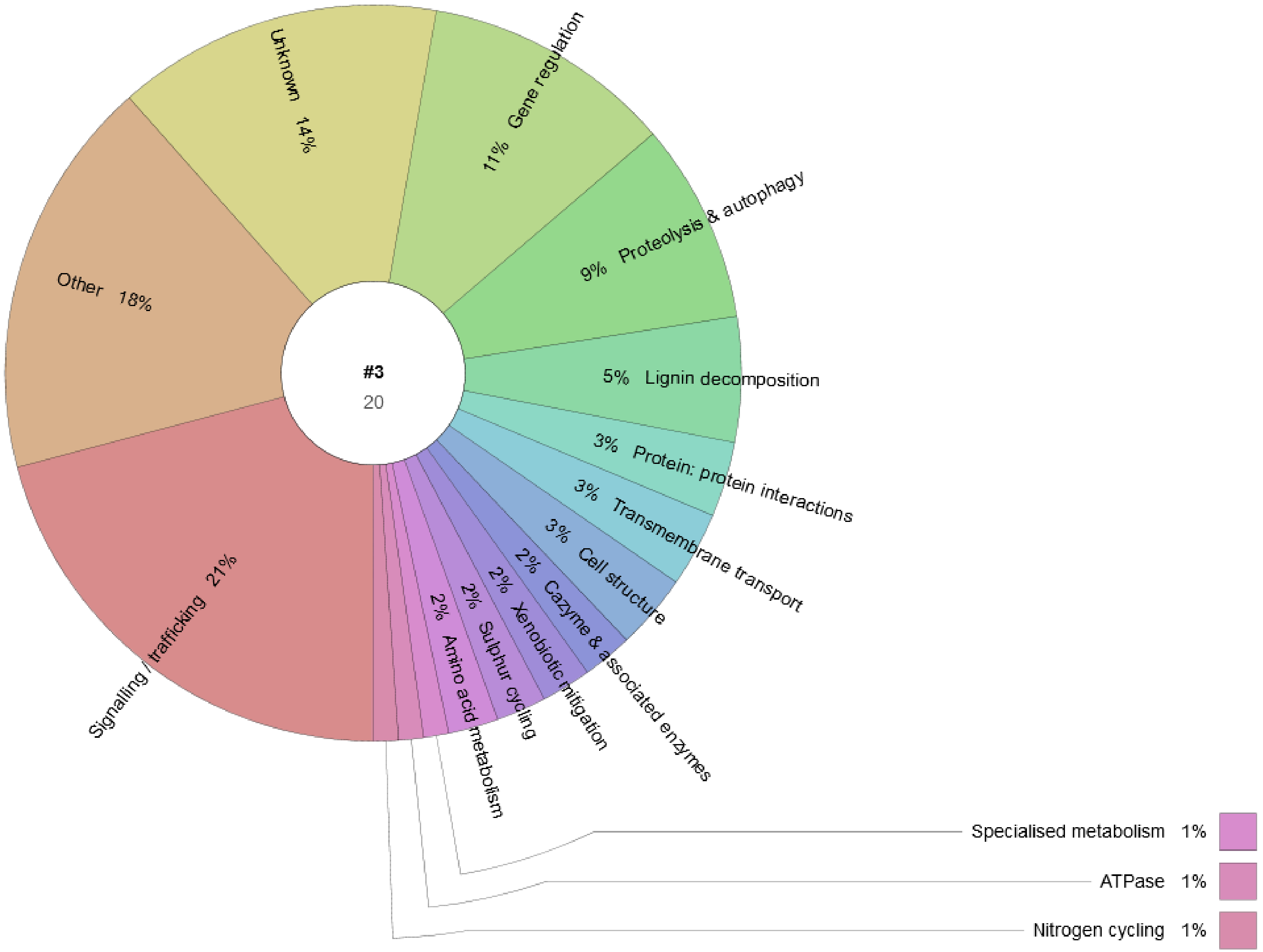

Figure S4. The functional proteome of sample #1 grown at 24°C.

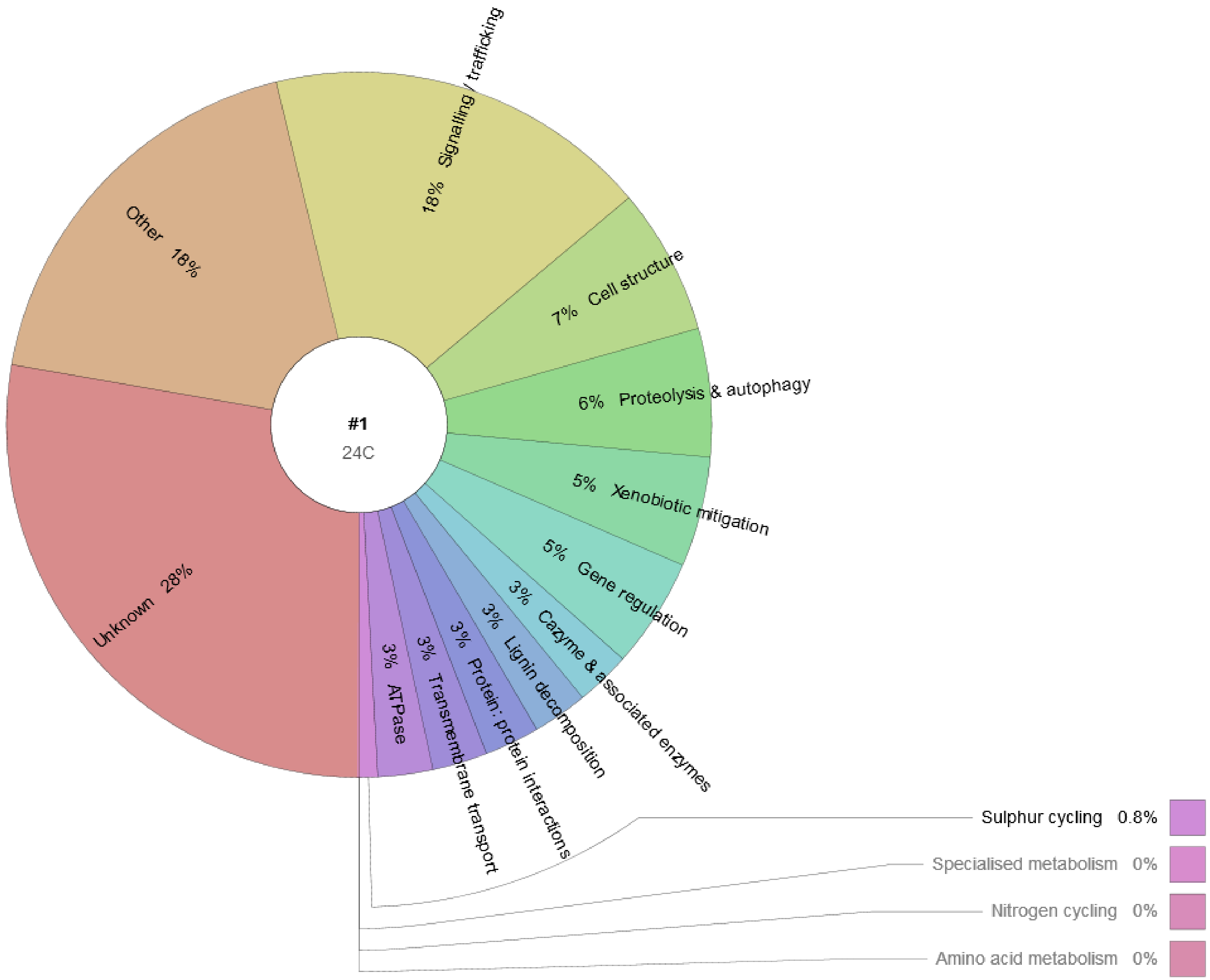

Figure S5. The functional proteome of sample #2 grown at 24°C.

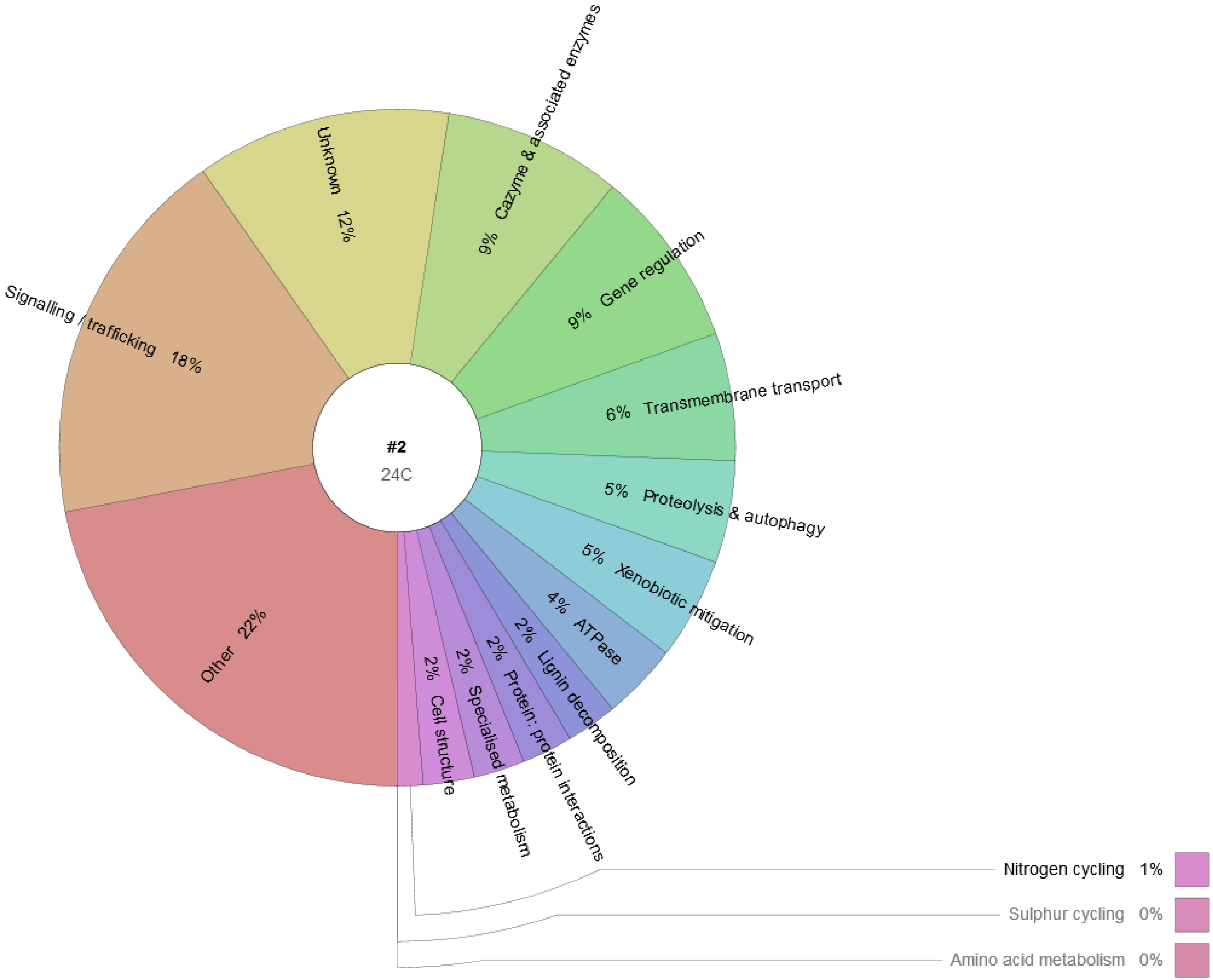

Figure S6. The functional proteome of sample #3 grown at 24°C.

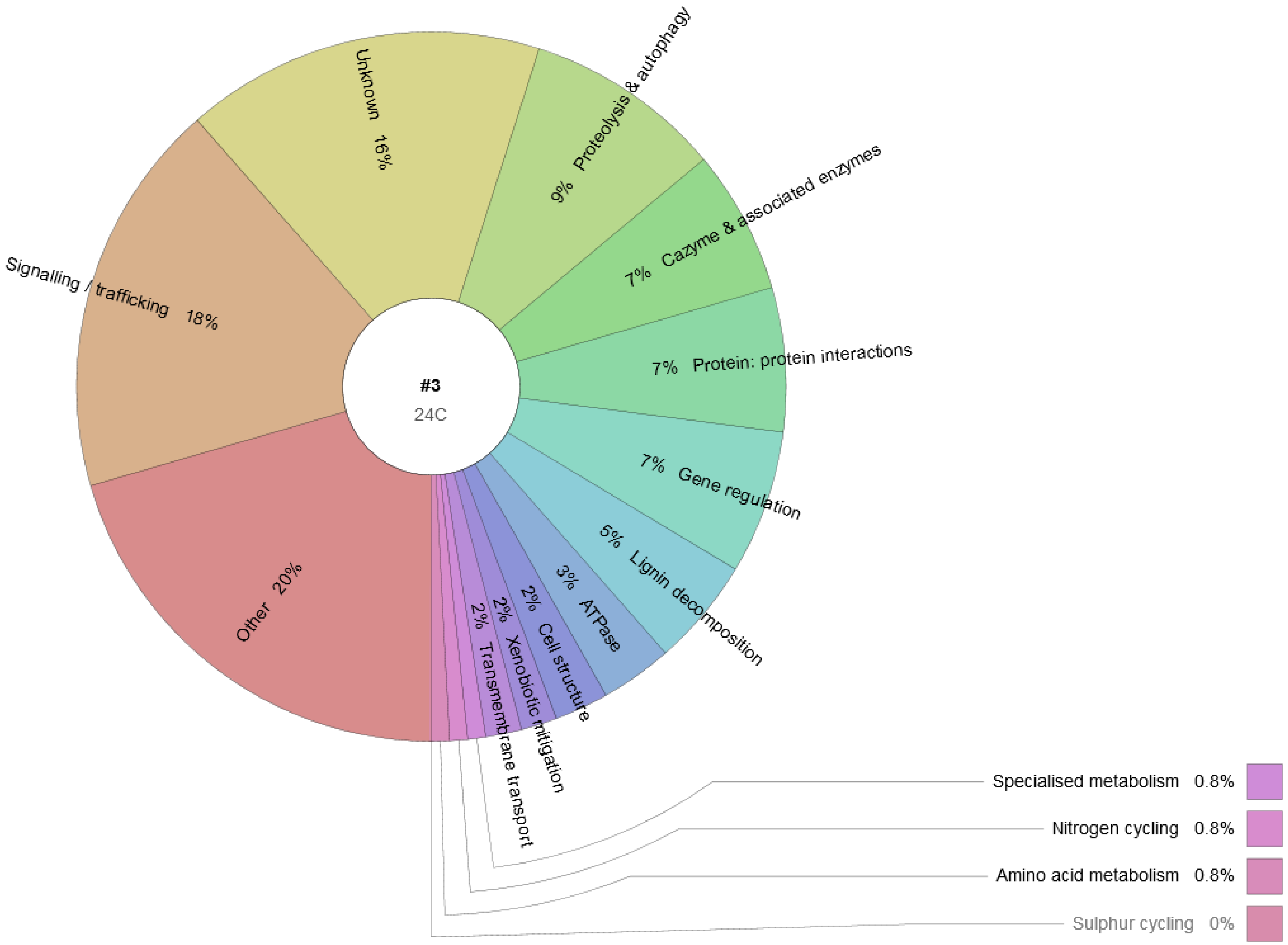

Supplementary table S4. The genomic locations of all ATPase domains identified, with characterisation in Figure 4 (main text). Blue text indicates they were identified in samples from 20°C, and red text indicates 24°C.

**Chaperone**	**Transmembrane transport**	**Signalling / trafficking**	**Nucleic acid processing**
Bjead1_1|116230|e_gw1.18.238.1	Bjead1_1|105428|e_gw1.3.1027.1	Bjead1_1|103129|e_gw1.1.35.1	Bjead1_1|26624|fgenesh1_pg.5_#_390
Bjead1_1|117003|e_gw1.20.7.1	Bjead1_1|32202|fgenesh1_pg.42_#_22	Bjead1_1|101452|e_gw1.1.962.1	
Bjead1_1|70747|estExt_fgenesh1_pg.C_110132	Bjead1_1|103761|e_gw1.2.514.1	Bjead1_1|165932|gm1.2371_g	
Bjead1_1|171119|gm1.7558_g	Bjead1_1|127082|estExt_Genewise1.C_2_t10346	Bjead1_1|79311|gw1.3.175.1	
Bjead1_1|107330|e_gw1.5.1089.1	Bjead1_1|30475|fgenesh1_pg.22_#_112		
Bjead1_1|70747|estExt_fgenesh1_pg.C_110132	Bjead1_1|28469|fgenesh1_pg.11_#_234		
Bjead1_1|25154|fgenesh1_pg.2_#_341	Bjead1_1|115643|e_gw1.17.334.1		
Bjead1_1|174536|gm1.10975_g			
Bjead1_1|173180|gm1.9619_g			
Bjead1_1|118830|e_gw1.25.108.1			

Supplementary information S1 - HPLC Mass Spectrometry method

HPLC-MS/MS was carried out using an ESI ion trap mass spectrometer, an LCQ DECA XP (ThermoFinnigan, Hemel Hempstead,UK). For the HPLC separation of the peptide sample, 7 µl (dissolved in 0.1% TFA in water) was injected into a C18 capillary pepmap column (250 mm*300 µm, Thermoscientific, UK) with a mobile phase flow rate of 4 µl/minute. The sample (5 µl) was injected into a mobile phase of 2% acetonitrile, 98% water (0.1% formic acid) with the elution mobile phase comprising 0.1% formic acid in acetonitrile. The 2% acetonitrile was maintained for 5 minutes before a gradient to 60% over 40 minutes was applied using a Dionex 3000 HPLC system (Thermoscientific, UK). The eluent was passed through a low flow electrospray needle and analysed utilising a spray voltage of 3 kV, a capillary voltage of 10 V, a capillary temperature of 185˚C as ion source values. Spectra were acquired in a positive, data-dependent acquisition mode in which the mass spectrometer first acquires a full scan mass spectrum between 475 and 2000 Da. The MS/MS spectra of the three most abundant ions in the spectrum were recorded using a collision energy of 35 arbitrary units. The next full scan spectrum was initiated. This process was repeated throughout the HPLC-MS/MS run with dynamic exclusion parameters excluding any ions whose MS/MS spectra were obtained three times from further analysis for 3 min. The resultant spectra were analysed on an in-house Mascot server.

Supplementary table S5. The genomic locations of all the proteins predicted to be involved in carbohydrate metabolism or lignin decomposition, as shown in Figure 4 (main text).

**Proteins identified at 20°C**	**Predicted function**	**Proteins found at 24°C**	**Predicted function**
Bjead1_1|117806|e_gw1.22.244.1	Glycoside hydrolase 31	Bjead1_1|171368|gm1.7807_g	Glycoside hydrolase 1
Bjead1_1|107427|e_gw1.5.989.1	Glycoside hydrolase 18	Bjead1_1|174107|gm1.10546_g	Glycoside hydrolase 3
Bjead1_1|123254|e_gw1.55.18.1	Polysaccharide lyase 6	Bjead1_1|26706|fgenesh1_pg.6_#_29	Glycoside hydrolase 16
Bjead1_1|28047|fgenesh1_pg.10_#_19	Galactokinase	Bjead1_1|117806|e_gw1.22.244.1	Glycoside hydrolase 31
Bjead1_1|103129|e_gw1.1.35.1	Galactokinase domain	Bjead1_1|171864|gm1.8303_g	Alpha-L-arabinofuranosidase
Bjead1_1|168388|gm1.4827_g	Short chain dehydrogenase	Bjead1_1|123254|e_gw1.55.18.1	Polysaccharide lyase 6
Bjead1_1|41582|fgenesh1_kg.17_#_199_#_Locus11735v1_medCvg15.6s	Copper radical oxidase	Bjead1_1|26422|fgenesh1_pg.5_#_188	Carbohydrate kinase
Bjead1_1|109531|e_gw1.7.863.1	Cupredoxin	Bjead1_1|133438|estExt_Genewise1.C_8_t20033	Major Intrinsic Protein Family
Bjead1_1|183533|estExt_Genemark1.C_130347	Tyrosinase	Bjead1_1|67475|estExt_fgenesh1_pg.C_1_t20179	Alpha amylase domain
Bjead1_1|34067|fgenesh1_kg.1_#_869_#_Locus2702v3_medCvg78.2s	Glyoxylate reductase	Bjead1_1|118657|e_gw1.24.388.1	Peroxidase
Bjead1_1|116751|e_gw1.19.59.1	Peroxidase	Bjead1_1|116984|e_gw1.19.139.1	Peroxidase
Bjead1_1|118535|e_gw1.24.48.1	Peroxidase	Bjead1_1|121766|e_gw1.37.43.1	Small peroxidase
Bjead1_1|116984|e_gw1.19.139.1	Peroxidase	Bjead1_1|25154|fgenesh1_pg.2_#_341	Citrate synthase domain
Bjead1_1|100659|gw1.24.421.1	Peroxidase	Bjead1_1|24287|fgenesh1_pg.1_#_234	Mannosyltransferase
Bjead1_1|121766|e_gw1.37.43.1	Small peroxidase	Bjead1_1|120730|e_gw1.32.72.1	Mannosyltransferase
		Bjead1_1|24243|fgenesh1_pg.1_#_190	Glycosyltransferase family 8

Supplementary table S6. The genomic locations of all the proteins predicted to be involved in specialised metabolism or xenobiotic mitigation, as shown in figures 5 and 6 (main text).

Proteins identified at 20°C	Predicted function	Proteins identified at 24°C	Predicted function
Bjead1_1|104989|e_gw1.3.831.1	Cytochrome P450	Bjead1_1|101126|e_gw1.1.445.1	Isopenicillin synthase
Bjead1_1|121105|e_gw1.34.180.1	Cytochrome P450	Bjead1_1|187109|estExt_Genemark1.C_360067	Cytochrome P450
Bjead1_1|112794|e_gw1.11.491.1	Terpenoid biosynthesis	Bjead1_1|100963|gw1.17.624.1	Cytochrome P450
Bjead1_1|25602|fgenesh1_pg.3_#_277	Fatty acid synthase	Bjead1_1|32434|fgenesh1_pg.49_#_8	Cytochrome P450
Bjead1_1|75451|gw1.13.24.1	NRPS	Bjead1_1|107364|e_gw1.5.324.1	Cytochrome P450
Bjead1_1|121952|e_gw1.39.80.1	Diketogulonate reductase	Bjead1_1|84823|gw1.36.55.1	Cytochrome P450
Bjead1_1|117162|e_gw1.20.234.1	Hydantoinase/ oxoprolinase	Bjead1_1|119120|e_gw1.26.116.1	Cytochrome P450
Bjead1_1|28502|fgenesh1_pg.11_#_267	Ca/Zn/Co efflux pump	Bjead1_1|107207|e_gw1.5.1492.1	Aldo keto reductase
Bjead1_1|24314|fgenesh1_pg.1_#_261	Reactive nitrogen intermediates (RNI)-like	Bjead1_1|24401|fgenesh1_pg.1_#_348	Aldo keto reductase
Bjead1_1|30817|fgenesh1_pg.25_#_48	Glutathione S transferase	Bjead1_1|29668|fgenesh1_pg.17_#_136	Aldo keto reductase domain
Bjead1_1|119732|e_gw1.28.56.1	Glutathione S transferase	Bjead1_1|106545|e_gw1.4.377.1	ETBE degradation
Bjead1_1|70747|estExt_fgenesh1_pg.C_110132	Thioesterase domain	Bjead1_1|70747|estExt_fgenesh1_pg.C_110132	Thioesterase domain
Bjead1_1|105267|e_gw1.3.846.1	Copper amine oxidase	Bjead1_1|115103|e_gw1.16.22.1	Thioesterase
Bjead1_1|119205|e_gw1.26.39.1	Rieske domain		
Bjead1_1|109592|e_gw1.7.749.1	Salicylate hydroxylase (domain)		
Bjead1_1|117143|e_gw1.20.23.1	Haloacid dehyalogenase		
Bjead1_1|119726|e_gw1.28.175.1	Prenyltransferase		

Supplementary table S7.

**Functional prediction**	**20°C**	**24°C**
Unknown	55	63
Carbon acquisition & mitigation	34	41
Proteolysis & autophagy	20	22
Protein: protein interactions	14	13
Signalling / trafficking	41	59
Xenobiotic mitigation	4	0
Specialised metabolism	5	3
Transmembrane transport	16	10
ATPase	7	10
Cell structure	6	13
Nitrogen cycling	4	2
Sulphur cycling	4	1
Gene regulation	19	21
Amino acid metabolism	7	1
Other	51	65
